# Retrocochlear Auditory Dysfunctions (RADs) and Their Treatment: A Narrative Review

**DOI:** 10.3390/audiolres16010005

**Published:** 2025-12-23

**Authors:** Domenico Cuda, Patrizia Mancini, Giuseppe Chiarella, Rosamaria Santarelli

**Affiliations:** 1 Department of Medicine and Surgery, University of Parma, 43121 Parma, Italy; 2 Guglielmo da Saliceto Hospital Piacenza, 29121 Piacenza, Italy; 3 Department of Sense Organs, Sapienza University of Rome, 00184 Rome, Italy; p.mancini@uniroma1.it; 4 Unit of Audiology, Phoniatrics and Vestibology, Regional Centre of Cochlear Implants and ENT Diseases, Department of Experimental and Clinical Medicine, Magna Graecia University, 88100 Catanzaro, Italy; 5 Department of Neurosciences, University of Padova, 35122 Padova, Italy; rosamaria.santarelli@unipd.it; 6 Audiology Service, Santi Giovanni e Paolo Hospital, 30122 Venezia, Italy

**Keywords:** sensorineural hearing loss, retrocochlear hearing loss, auditory neuropathy, auditory neuropathy spectrum disorders, ANSD, auditory processing disorder, neural desynchronization, genetic mutations, assistive listening device, hearing aids, cochlear implant, auditory training, hidden hearing loss, retrocochlear auditory dysfunctions, RAD

## Abstract

**Background/Objectives**: Retrocochlear auditory dysfunctions (RADs), including auditory neuropathy (AN) and auditory processing disorders (APD), encompass disorders characterized by impaired auditory processing beyond the cochlea. This narrative review critically examines their distinguishing features, synthesizing recent advances in classification, pathophysiology, clinical presentation, and treatment. **Methods**: This narrative review involved a comprehensive literature search across major electronic databases (e.g., PubMed, Scopus) to identify and synthesize relevant studies on the classification, diagnosis, and management of AN and APD. The goal was to update the view on etiologies (genetic/non-genetic) and individualized rehabilitative strategies. Diagnosis relies on a comprehensive assessment, including behavioral, electrophysiological, and imaging tests. Rehabilitation is categorized into bottom-up and top-down approaches. **Results**: ANSD is defined by neural desynchronization with preserved outer hair cell function, resulting in abnormal auditory brainstem responses and poor speech discrimination. The etiologies (distal/proximal) influence the prognosis for interventions, particularly cochlear implants (CI). APD involves central processing deficits, often with normal peripheral hearing and heterogeneous symptoms affecting speech perception and localization. Rehabilitation is multidisciplinary, utilizing bottom-up strategies (e.g., auditory training, CI) and compensatory top-down approaches. Remote microphone systems are highly effective in improving the signal-to-noise ratio. **Conclusions**: Accurate diagnosis and personalized, multidisciplinary management are crucial for optimizing communication and quality of life. Evidence suggests that combined bottom-up and top-down interventions may yield superior outcomes. However, methodological heterogeneity limits the generalizability of protocols, highlighting the need for further targeted research.

## 1. Introduction

Sensorineural hearing loss (SNHL) has traditionally been divided into cochlear and retrocochlear forms, based on the site of the lesion: the former affects the cochlea, while the latter involves the auditory nerve and central auditory pathways [1]. Unlike cochlear hearing loss (HL), retrocochlear forms were classified as exhibiting specific audiological features, such as preserved cochlear function in audiometry testing, markedly altered auditory brainstem responses (ABR), and speech perception impairment that exceeds what would be expected based on pure tone thresholds [2]. It is currently recognized that various retrocochlear pathologies present with more complex audiological phenotypes. From a neuroradiological perspective, lesions were classified as retrocochlear, whereas those without neuroradiological correlates were ascribed to auditory processing disorders or defined as “central”. Recent advances in neurophysiology, genetics, and neuroimaging necessitate an ongoing re-evaluation of this topic.

Considering these developments, it is proposed that we adopt the more inclusive term retrocochlear auditory dysfunction (RAD) instead of “retrocochlear hearing loss”. RAD encompasses two main categories: auditory neuropathy (AN) [3,4], in which the lesion involves the auditory nerve, and auditory processing disorder (APD) [5,6], which primarily affects the central auditory pathways.

AN represents the paradigm of RAD: patients show nearly normal (OHC) function (demonstrated by intact otoacoustic emissions), but present with marked morphological and threshold abnormalities in ABR and poor speech discrimination [7]. The hallmark of AN is neural desynchronization, which impairs the transmission of complex signals such as speech [8].

APDs are also characterized by neural desynchronization, which is associated with deficits in other perceptual components, such as the ability to segregate acoustic sources. In these cases, ABR alterations are less apparent.

Excluded from the definition of RAD is cochlear synaptopathy—exemplified by otoferlin mutation, which causes the dysfunction of inner hair cell ribbon synapses [9,10,11]—as it represents a purely endocochlear pathology [12,13]. Similarly, so-called “hidden hearing loss” (HHL) [14], which is frequently associated with noise exposure, aging, or peripheral neuropathies [15], will be discussed but is not included in this conceptualization because the underlaying mechanisms are difficult to categorize and are generally related to peripheral synaptic damage [16]. Evidence suggests that noise and aging are also associated with central auditory system damages [17,18] and can therefore present both cochlear manifestations as well as patterns that are characteristic of neuropathy and APD. Conversely, post-synaptic pathologies—such as those due to mutations in the OPA gene [19,20]—are included among auditory neuropathies, as the lesion affects the auditory nerve and typical patterns of neural desynchronization are observed [11].

In summary, the distinction between cochlear and retrocochlear HL is evolving due to scientific advances, allowing for a broader perspective of retrocochlear auditory dysfunctions that includes both principal subtypes of AN and APD while excluding certain specific forms of synaptopathy and HHL.

The aim of this narrative review is to critically examine the distinctive features of RADs (AN and APD) and the most recent advances in their treatment. The review involved a comprehensive literature search across major electronic databases (e.g., PubMed, Scopus, Cochrane). In addition to databases, the bibliography was supplemented with material that was available from books and institutional websites. The research covered the last three decades up to the current year, although to reconstruct the ‘historical’ aspects of certain nosological frameworks, seminal studies from earlier periods were also cited.

## 2. Auditory Neuropathies (AN)

The term AN was introduced by Starr et al. (1991, 1996) [3,4] to describe a hearing dysfunction characterized by damage to the auditory nerve with preservation of OHC function (evidenced by the presence of otoacoustic emissions, OAE, or cochlear microphonics, CMs). Alterations of auditory nerve function result in a decrease in the auditory input to the CNS, as well as in the dys-synchrony of individual fiber discharge, leading in turn to the absence or severe abnormalities of ABR and speech perception impairment. On the other hand, damage to the afferent compartment of the auditory periphery at any level, including inner hair cells and synapses, may also result in the disruption of the temporal coding of acoustic signals in the auditory fibers. Therefore, the term AN encompasses a multiplicity of manifestations, ranging from mild difficulties in hearing in noisy environments to profound deafness. In 2008, a panel of experts [21] expanded and redefined the term as auditory neuropathy spectrum disorder (ANSD) to reflect the complexity and variability of the disorder and to unify the terminology for the various clinical presentations, due to different etiological mechanisms.

In recent decades, genetic studies and the analysis of lesion sites correlated with clinical data have helped to clarify the underlying molecular mechanisms, thereby improving screening, assessment, and therapeutic strategies. Evidence of a dichotomy in cochlear implant (CI) outcomes in ANSD—like cochlear deafness in some cases and unsatisfactory results in others—has contributed to a more precise classification, together with the molecular characterization of synaptopathies and neuropathies, resulting in a possible ‘topodiagnostic’ and therefore prognostic mapping of many neuropathies [19,20,22,23,24,25,26,27,28,29,30,31,32]. Genetic advances and post-mortem evaluation of the eighth nerve have made it possible to identify specific post-synaptic alterations involving the neural component of the synapse and to include them among auditory neuropathies. This allows for the distinction between distal (or dendritic) neuropathies, which affect the terminal axon (or dendrite) and are associated with a good prognosis with CI, and proximal (or axonal) neuropathies, which involve the central axon and cell body and result in a poor outcome with CI use. This distinction is because distal neuropathies partially preserve the neuronal interface for electrical stimulation, whereas proximal neuropathies compromise its effectiveness. Table 1 provides a comprehensive overview of the mechanisms, clinical presentations, and genetic factors associated with distal and proximal auditory neuropathies, together with the relevant bibliographical references.

The detailed etiological and topodiagnostic classification into distal (dendritic/post-synaptic) and proximal (axonal/somatic) neuropathies is of crucial importance, as the site of the lesion significantly influences the prognosis for rehabilitation, particularly with CI. From a functional perspective, the primary effect of ANSD is neural dys-synchrony, which impairs the temporal transmission of acoustic signals. Clinically, this translates into a severely reduced ability to perceive and decode speech, especially in noisy environments: a deficit that often appears to be disproportionate to the pure tone audiometric thresholds. In summary, Table 1 highlights the heterogeneity of auditory neuropathy, underscoring the role of diverse genetic mutations and non-genetic insults in the pathogenesis and clinical spectrum of this disorder. From a clinical perspective, ANSD can present at any age, though a significant proportion of cases are identified in the neonatal period (often associated with risk factors like hyperbilirubinemia, prematurity, or hypoxia) or early childhood. In the clinic, the primary symptom that should raise suspicion of ANSD is a speech perception deficit that is disproportionate to the pure tone audiometric threshold. Clinicians must be particularly aware of congenital, progressive, or fluctuating hearing loss in a child who fails to develop language appropriately, or an adult complaining of significant difficulty understanding speech, particularly in noise, despite normal or near-normal audiometric thresholds. The absence of auditory brainstem responses (ABR) coupled with the presence of otoacoustic emissions (OAE) or cochlear microphonics (CMs) is the pathognomonic electrophysiological sign for diagnosis. Given the high genetic heterogeneity (Table 1), the presence of associated neurological signs (e.g., peripheral neuropathy, ataxia, ophthalmoplegia) or a familial history of hearing loss should immediately trigger a referral for genetic counseling and diagnostic workup, to guide prognosis and rehabilitation strategies and inform family planning.

## 3. Auditory Processing Disorders (APD)

Brainstem dysfunctions can be associated with APD, impacting the brain processing of auditory information. APD is a broad term which originally linked to the bi-hemispheric involvement of the temporal lobe. Subsequently, it has been extended to other cortical and subcortical structures, and finally, to the unilateral or bilateral of auditory structures within the midbrain [5]. Distinct auditory processing impairments may result from dysfunction at the level of the brainstem. The audiological presentation varies according to the anatomical site of the lesion: damage to the pontomedullary junctions typically leads to varying degrees of HL, whereas lesions affecting the pons and inferior colliculi are generally not associated with HL, but rather with deficits in interaural and temporal decoding [80]. An increasing body of knowledge has further identified the brainstem as having a primary role in verbal perception and language development [81,82].

In summary, the spectral and temporal properties of complex sounds, as well as their localization, are processed by neural networks located in the brainstem; this initial phase of processing contributes to selectivity towards biologically relevant sounds in higher-order cortical networks (for a comprehensive review on this subject, see Felix et al., 2018) [83].

From a psychoacoustic perspective, every sound in the environment is characterized by specific perceptual aspects, such as direction of origin, pitch, timbre, temporal profile, etc. These features are decomposed and extracted into different neural streams, starting from the auditory nerve up to the auditory cortex. Some of these features and their involvement at the various stages of the auditory pathway are reported in Table 2, adapted from Felix et al. (2018) [83], and represent specialized streaming. In the brainstem, this information is processed through the mechanisms of grouping and segregation of auditory features.

It is noteworthy that, already, much of the feature extraction that is observable in the cortex has already occurred at the level of the inferior colliculi. Different networks within the brainstem are specialized in recoding information related to both identity and origin of complex sounds. These attributes, together with selective attention, are essential for understanding speech in acoustically challenging environments. Alongside the ascending (bottom-up) system, a significant role is played by the descending (top-down) modulations exerted by the cortex at virtually all levels of the auditory pathway [84].

Finally, all this information is integrated at the level of the auditory cortex, where auditory images and the entire acoustic scene are reconstructed [85,86,87,88].

APD may occur due to a variety of causes, from lack of synchronicity of the auditory fibers to lesions occurring at any site involving the pontomedullary junction to the roof of the inferior colliculi, therefore representing a variable combination of the above-mentioned auditory processing functions.

The lack of a clear definition as to what constitutes APD led to a few task forces (ASHA, 2005 [6]; AAA, August 2010 [89]; BSA, 2018 [90]) convening to develop consensus statements and establish best practice principles related to its diagnosis and management. APD manifests itself primarily in the auditory system and predominant complaints are auditory, depending upon the site of the lesion, and might involve neurophysiological functions such as sound localization, auditory frequency discrimination, temporal sound processing, pattern processing, dichotic listening, and performance in competing or degraded signals.

ASHA [6] defines APD as a deficit in the neural processing of auditory stimuli that may coexist with, but is not the result of, dysfunction in other modalities. Nevertheless, as auditory perception language and cognitive skills (e.g., attention, working memory, and short term memory, to mention a few) are closely related during development, APD may lead to or be associated with difficulties in higher order language, learning, and communication function [6,91]. Knowledge of these mechanisms is the basis for understanding and improving APD diagnosis and to develop individualized interventions to address specific deficit areas [92].

The symptoms referred by adults and children are multiple and regard both auditory and communicative, as well as behavioral, domains. A broad, although not exhaustive, list is as follows: difficulty in discriminating speech in quiet but more often in noise and reverberating acoustic environment; problems with the ability to localize the source of a signal; difficulty hearing on the phone; inconsistent or inappropriate responses to requests for information; difficulty following rapid speech, frequent requests for repetition and/or rephrasing of information; difficulty following directions; difficulty or inability to detect the subtle changes in prosody that underlie humor and sarcasm; difficulty learning a foreign language or novel speech materials, especially technical language; difficulty maintaining attention; a tendency to be easily distracted; poor singing, musical ability, and/or appreciation of music; and academic difficulties, including reading, spelling, and/or learning problems [89].

The diagnosis of APD is based on behavioral and electrophysiological tests that assess complex auditory abilities, such as understanding speech in the presence of noise, dichotic listening, and temporal processing [93]. The use of advanced imaging techniques, such as magnetic resonance imaging (MRI) and diffusion tensor imaging, together with specific auditory processing tests, is essential to detect possible central lesions and assess their impact on perceptive abilities [94,95].

APD predominantly begins in childhood, and it is very common among individuals with learning difficulties, such as dyslexia, attention deficit/hyperactivity disorder (ADHD), and autism spectrum disorders (ASD). In patients with APD, the anatomy and function of the external, middle, and inner ear are normal; however, deficits emerge in the neural processing of acoustic stimuli that cannot be attributed to higher-order cognitive or linguistic disorders, and the disorder may also be present in the adult population.

The actual prevalence of APD is not easily quantifiable, as it is strongly influenced by the diagnostic criteria used. According to Wilson and Arnott (2013) [96], applying nine different sets of diagnostic criteria to a population of children evaluated for suspected APD, the prevalence ranged from 7.3% when using very restrictive criteria to 96.0% with more inclusive criteria. Even among adults over the age of 55, the prevalence of APD appears significant, varying between 27% and 65%, while in those over eighty, it can reach up to 95% [97,98]. However, considerable caution is warranted when interpreting these percentages, as—regardless of methodological issues (definitions, normative data, etc.)—the adult population encompasses a multitude of factors that do not necessarily indicate APD (such as noise-induced synaptopathies, sociocognitive aspects, etc.). Nevertheless, these findings suggest that the issue is likely currently underestimated.

These data underline the importance of greater standardization and the development of shared guidelines for the diagnosis and management of APD.

The causes and risk factors associated with these disorders are multiple and well-documented in the literature [99]. Table 3 provides an up-to-date and evolving overview of these elements. It summarizes the risk factors, clinical presentations, and mechanisms underlying APD in children and adults. Despite the brainstem’s central role in auditory function, depending on the cause and site of the lesion, symptoms might be silent or overlooked. In adults, the auditory symptoms are shadowed by a variety of co-present neurological deficits [80]. Up to 50% of subjects with brainstem lesions do not complain of auditory disorders, although they are present (e.g., in multiple sclerosis) [100], contributing to the uncertainty of the prevalence of symptoms [80]. Clinical presentations and mechanisms vary, including functional deficits, fluctuating auditory disorders, tinnitus, and difficulties in noisy environments. The complexity and heterogeneity of APD highlight the contributions of both the peripheral and central mechanisms, as well as the genetic, developmental, and acquired factors.

Some special considerations are required for APD isolated forms in adults, as although they likely result from peripheral and central multi-site damage and therefore represent cases of uncertain interpretation, they probably highlight an area of unmet health needs.

An interesting cross-sectional population study, conducted in Finland by Hannula et al. (2011) [107], investigated the association between self-reported hearing problems and measured hearing thresholds in a random sample of 850 individuals aged between 54 and 64 years. The prevalence of self-reported hearing problems was 37.1% (“hearing difficulties”), 43.3% (“difficulty following a conversation in the presence of noise”), 29.2% (“tinnitus”), and 17.2% (“hyperacusis”). More than half of the subjects had normal hearing in the better ear (PTA4 kHz < 20 dB HL). Therefore, self-reported hearing difficulties appear to be more common than HL, as defined by audiometric measurement.

Spankovich et al. (2018) [108], in the United States, analyzed the prevalence and determinants of self-reported hearing difficulties (HD) and tinnitus in individuals with normal audiometric thresholds. The study included 2015 participants aged between 20 and 69 years with normal audiometric thresholds in both ears (PTA4 ≤ 25 dB HL). Fifteen percent reported HD, and 10.6% reported persistent tinnitus. Factors associated with an increased likelihood of HD included tinnitus; balance problems; noise exposure; arthritis; visual difficulties; neuropathic symptoms; and physical, mental, or emotional issues. The study demonstrates that the prevalence of HD is contingent upon the specific audiometric criteria employed to define “normal hearing.” Furthermore, it underscores that HD may originate from both peripheral and central auditory pathway alterations, as well as from extra-auditory factors, including psychological health, environmental noise exposure, and comorbid medical conditions.

Even when applying a more stringent audiometric criterion (hearing threshold < 15 dB at all frequencies in both ears), the prevalence of self-reported HD in the United States remains significant [15]. From a cohort of 2783 participants, the authors identified 682 individuals with normal hearing (aged 21–67 years). Among these, 12% reported HD, corresponding to an overall prevalence of 2.9%. No significant differences were found in audiological tests between those who reported HD and those who did not. However, associated risk factors included noise exposure, depression, visual impairment, neuropathy, and low income. These findings suggest that HD can result from both audiological and non-audiological causes, such as psychological and environmental factors.

A different perspective on the association between the perception of hearing difficulties and audiometric thresholds involves investigating whether individuals with documented HL may perceive their own hearing as “normal”. Curti et al. (2019) [123] explored this topic in a cohort in which 1373 participants had HL, defined by audiometric thresholds greater than 25 dB HL. Of these, 68.5% reported having good hearing, despite audiometric evidence of HD. Younger age, female sex, belonging to ethnic minorities, better general health, and diet were associated with the self-perception of good hearing. Conversely, the presence of tinnitus, noise exposure, neuropathic symptoms, arthritis, and medication use were linked to a lower likelihood of perceiving good hearing.

The severity of HL affects perception: as stricter criteria are applied to define hearing loss, the proportion of individuals reporting good hearing declines. The authors note that mild HL frequently goes unnoticed, which may lead to delays in both diagnosis and intervention. Additionally, social and cultural factors can influence how HL is perceived.

Beck et al. (2019) [109] published a review focusing on HD and speech perception in noisy environments observed in adults with normal hearing thresholds, a group estimated to be around 26 million in number in the United States. These symptoms may result from conditions such as cochlear synaptopathy (HHL) but are mainly attributable to the broader group of APD, including so-called central presbycusis, the consequences of head trauma, and other factors. Diagnosis of these disorders requires specific tests, which are rarely employed in clinical practice.

## 4. RAD Rehabilitation

RAD diagnosis and intervention must be carried out by multidisciplinary teams, who determine the most appropriate and individualized therapeutic approach (auditory training and compensatory strategies, low gain HAs or CI, remote microphone system, environmental acoustic modifications) and/or whether additional intervention (cognitive training, behavioral communication strategies, metacognitive strategies) is indicated. Rehabilitative approaches have been classified by ASHA (2005) [6] and the AAA (2010) [89], according to the main neurophysiological process in bottom-up and top-down interventions. Typically, a bottom-up intervention involves auditory training (AT), hearing devices, and assistive listening systems to improve signal quality and therefore transmission of sounds to the central nervous system. Top-down interventions make use of compensatory strategies, teacher/speaker adaptation, and games strategies, activating cognitive and language skills to compensate for a poor bottom-up decoding to improve communication.

A successful therapeutic approach, besides being individualized and accessible, should also emphasize patient motivation and self-efficacy to foster adherence to the treatment plan. A low compliance rate also makes it difficult to draw conclusions regarding the efficacy of therapeutic protocols [6,124].

### 4.1. Bottom-Up Rehabilitative Approaches

#### 4.1.1. Auditory Training in APD

AT is a bottom-up rehabilitative approach which is aimed at specifically addressing the function of the involved auditory process. AT requires the identification of the specific auditory deficits through testing and patients’ history, leading to treatments that are deficit specific [6,125]. A typical rehabilitative paradigm is a listening task which is not dissimilar to the one the patient shows difficulty with, and training involves exercises of speech perception in noise, monoaural low-redundancy speech, lateralization, temporal intensity and frequency discrimination, and bilateral interaction/integration [126]. Often AT must incorporate different non-auditory approaches to better meet individual needs, and this complexity, together with the variability of age at diagnosis and cause of APD, has limited the classification of outcomes from a medicine-based evidence point of view [127]. Further, while for children and adolescents APD is more often the result of congenital abnormal hearing pathway development, in adults it is more often the consequence of vascular and traumatic injuries, making it difficult to separate AT efficacy from spontaneous recovery [128].

The basic principle of AT relies on neural plasticity. In the auditory system, plastic changes are a result of neuronal responses to both external stimuli and internal stimuli, because of different processes such as developmental, compensatory to lesion, or learning-related plasticity [129]. AT, being a complex task, is more likely to benefit from plasticity through brain reorganization mechanisms such as the activation of neurons and neural connections that were previously in a state of rest, or formation of new connections. These adaptive changes take place to different degrees, according to subject age, and have been documented with cortical auditory-evoked potentials with either passive or active discrimination paradigms (mismatch negativity and P300 amplitudes) [130,131]. More recently, in contrast with the concept of auditory brainstem nuclei as passive relay stations for behaviorally relevant signals, a new theoretical framework has been proposed, based on animal research, which supports the presence of subcortical auditory regions by means of local reorganization, contextual, and experience-dependent modulations, which can all influence subcortical auditory processing [132].

Concerning the age issue, in the mature, typically developed auditory system, there is a general reduction in bottom-up plasticity and a greater emphasis on top-down mechanisms [133]. Nevertheless, brain plasticity has been clinically documented in older adults by means of MRI structural and connectivity changes in all subgroups of subjects who underwent AT, but more so when AT was administered in combination with cognitive training [134]. This outcome is supported by research on the inferior colliculus’ role in the attentional processing mechanisms (e.g., negative plasticity involved in tinnitus perception) [135]. Also, especially in elders, the cognitive role in auditory processing has been further conceptualized under a theoretical framework where attention and memory integrate hearing when comprehending and communicating in realistic situations [136].

AT can be delivered as formal or informal training. Formal training often requires the use of commercially available computer software (computer-based auditory training, CBAT), usually targeting specific auditory processing (e.g., dichotic listening, weaker ear speech in noise training) by using adaptive techniques to improve processing in a graded manner with an intensive training schedule [137]. Informal AT is considered to be cost effective, although research reports are mainly single-case, and it lacks an evidence-based approach. For both formal and informal training, there is a summary of rules that support training effectiveness [127,138]. AT must include the skills that have been measured as altered. AT is time-consuming to develop and to maintain changes over time. Once again it stressed how commitment to regular training is important. To increase compliance, stimuli must be varied and engaging and difficulties must be progressive. It is considered that a correct score should be no less than 30% and no more than 70% to avoid floor and ceiling effects. Difficulty must be graduated as a function of patients’ performance improvements. For young children, the use of interactive games is effective and the association of other sensory inputs to the auditory stimuli is needed. Multisensory stimulation, engaging formats, feedback, and reinforcement facilitate home-based intensive training. Training typically focuses on activities such as temporal processing, dichotic processing, binaural integration/separation, language decoding and separation, auditory closure, discrimination, and interhemispheric transfer training, which addresses binaural hearing and binaural processing, such as interaural timing and intensity differences [139,140]. Auditory closure activities are integrated into AT for individuals with deficits in monaural low-redundancy speech. The purpose is to assist the individual in learning how to fill in the missing parts to perceive a meaningful whole. Temporal patterning and prosody training aims to improve the recognition and use of prosodic patterns of speech. Auditory discrimination training of isolated phonemes and phonemes in syllables and words can be carried out with formal and informal training in individuals with associated difficulty in phonological and phoneme awareness. Informal dichotic listening training includes activities such as informal speech in noise training, listening to lyrics of songs, listening with the weak ear to something of interest, and localization training in quiet and in noise.

The available CBAT software has mainly been developed in the English language. Computer programs can improve many of the above-mentioned aspects. CBAT can better determine the size of the progression steps and the size of the success-to-failure ratio to maintain patients’ motivation throughout feedback and reinforcement. Further computer programs have the structure for designing prospective and retrospective research aiming to measure the scientific evidence for different diagnostic and therapeutic protocols. An example of widely used CBAT are ARIA [141] or Fast ForWord [142], which uses AT protocol, while other programs such as Brain Fitness [143] and Earobics [144] are designed to carry out AT cognitive and/or language training, given the role of auditory attention and memory in speech perception [136].

Formal or informal AT is strongly dependent on stimulation and practice, which is further reflected in behavioral changes [137]. In this regard, one important research issue is whether the transfer of learning between different tasks is effective [126]. One criticality of bottom-up AT is its connection to diagnostic battery tests in use. Completeness of diagnosis is expected to define the subject’s strength and weakness to better address auditory deficits. Research in AT-induced learning transfer has shown how within-task (learning obtained in a task with a stimulus is generalized to a similar one) is possibly more beneficial than between-task (learning transferred to a completely different type of task and stimuli) and it is generally speculated that a process-specific training battery is more effective than a non-specific training battery [145], once again stressing the role of a complete diagnosis. Non-specific training is nevertheless important in all subjects where specific issues cannot be isolated. This last type of training is expected to recruit cognitive skills such as attention, which could facilitate the transfer of learning from perceptual discrimination tasks to language development [146].

The role of AT and subsequent rehabilitative strategies differs significantly between ANSD and APD, mirroring the differences in the site of the lesion. For patients with APD, AT is the cornerstone of rehabilitation. These programs utilize structured, intensive listening exercises (e.g., dichotic listening training, temporal processing tasks) aimed at improving the central nervous system’s ability to process auditory information. This approach directly addresses the underlying central deficit, offering significant benefits. Conversely, for individuals with AN, traditional AT is generally not the primary intervention and may be ineffective. The primary deficit in ANSD lies in the neural transmission, which AT cannot typically resolve. Rehabilitation for ANSD focuses primarily on restoring the input signal through CI, especially in cases of proximal/axonal involvement or the use of HAs with wide-band or digital processing capabilities when the residual function is preserved. Compensatory strategies (CS) (e.g., environmental modifications, visual cues, whole body listening techniques) are beneficial for both conditions, serving to mitigate the impact of the perceptual deficits in real-world settings.

#### 4.1.2. AT and Evidence-Based Approaches

The issue of evidence-based therapeutic approaches has been the subject of few randomized trial and systematic reviews. Often, studies selected for systematic reviews adopted mixed auditory, cognitive, and/or CS, making it difficult to isolate the specific factors contributing to treatment outcomes. This is especially true when language training is involved, owing its influence on auditory skills [147]. A randomized control trial (RCT) was published by Putter-Katz et al. (2008) [148]. The APD management approach was integrative and included top-down and bottom-up strategies. It focused on environmental modifications, remediation techniques, and CS. Training was conducted with monosyllabic and polysyllabic words, sentences, and phrases, in quiet and in noise. Comparisons of pre- and post-management measures indicated an increase in speech recognition performance in background noise and competing speech for the treatment group. Fey et al. (2011) [149] evaluated the effect of auditory and/or language intervention in subjects diagnosed with either single (ADD or language) or combined deficits through a systematic review. Research reporting on outcomes of auditory or language intervention in ADP disorders resulted in overall weak evidence of intensive short-term AT. The six studies found were mainly case series exploratory studies reporting auditory or auditory/neurophysiological outcomes. On the contrary, a prospective randomized controlled trial combining CABT bottom-up auditory training, an assistive listening device (FM), and top-down language training [150] found significant differences after intervention for several auditory and language measures for the study group only. Seemingly, another study [151] found a short-term significant benefit of CBAT in the intervention group only, as opposed to a control group who received a treatment based on CS and environmental modification (EM) at school and/or at home. Very few studies addressed the efficacy of AT alone or in combination with adjunctive procedures in adults with APD. Crum et al. (2024) [152] published a systematic review and meta-analysis that was drawn to evaluate evidence-based intervention, including AT, low-gain HAs and assistive listening devices. Seven out of sixteen selected studies looked at the effect of AT, by means of non-formal (singing, direct rehabilitation) and formal home-based computer-based auditory and cognitive training. Studies showed high methodological heterogeneity that made a meta-analysis impossible. Four out of six studies showed significant monoaural low redundancy outcomes post-training, while two out of six could not rule out the effect of test–retest training on the outcomes. Conclusions from the systematic review are mixed, mainly driven by methodological differences and patients’ low compliance to home-based training, once again underlining the need for further research with greater methodological strength, to address the efficacy of each AT therapeutic protocol to ascertain the length of therapy and whether periodic training is needed.

#### 4.1.3. Hearing Aids

Prescription HAs are personalized amplification devices fitted by audiologists or hearing instrument specialists. They represent the main intervention for most sensorineural cochlear hearing losses. Most research on HAs centers on mild-to-moderate peripheral hearing loss, consistently demonstrating the clear benefits for quality of life, listening ability, and communication [153,154,155,156]. When it comes to RAD, however, outcomes with amplification are mixed. Although making sounds louder can help with awareness and localization, speech understanding often remains a major challenge due to underlying neural processing issues. It is important to distinguish the potential applications across the two main diagnostic categories of RAD: ANSD and APD. While subjects with ANSD typically present with raised audiometric thresholds, those with APD generally exhibit thresholds that fall within the normal range.

#### 4.1.4. Hearing Aids in ANSD

ANSD represents a disorder encompassing various conditions characterized by the desynchronization of auditory nerve firing. In these cases, clinical observation points to deficits in specific temporal processing domains, such as low-frequency perception (coded primarily by temporal cues), temporal integration, gap detection, frequency modulation detection, and processing of interaural temporal differences. In contrast, intensity processing tends to remain relatively preserved.

AN is identified most in children due to universal hearing screening with AABR (automatic auditory brainstem response), but it can also appear in adults. It accounts for up to 10% of new pediatric hearing loss cases [157]. In adults, diagnosis usually occurs during evaluation for peripheral neuropathies or after failed HA fittings [158].

Children diagnosed with ANSD demonstrate perceptual profiles that differ from those observed in SNHL [159,160]. Individuals with ANSD tend to have challenges with temporal coding of auditory information, whereas cochlear deafness is characterized mainly by difficulties in frequency resolution, with temporal processing remaining relatively intact. Temporal dysfunction affects the temporal resolution, amplitude modulation detection, and temporal aspects of frequency discrimination, such as phase locking. The extent of temporal disruption is directly related to the degradation of speech perception. For these reasons, acoustic amplification has long been considered to be a less effective solution than cochlear implantation in cases of ANSD; HAs can increase the speech intensity but may also introduce distortion, whereas CI are thought to promote greater synchrony in neural firing. This consideration is particularly relevant in cases with severe HL, while it applies differently to moderate HL. ANSD presents with a broad variability in hearing thresholds. According to neurophysiological principles [161], increasing the sensation level through amplification could enhance neural synchrony, although this effect is not consistently observed in all cases of ANSD.

An expanding body of evidence informed the initial consensus statement regarding this complex topic, indicating that after comprehensive assessment of hearing loss severity, a properly supervised HA trial should be considered to be the primary intervention for pediatric patients with ANSD [21].

For instance, Rance et al. (2002) [162] demonstrated that approximately 50% of children with ANSD exhibit a significant improvement in open-set speech perception when utilizing amplification. In these situations, behavioral audiograms do not reliably predict speech perception outcomes. However, cortical event-related potentials (ERPs) can offer prognostic value: children displaying age-appropriate ERPs tend to benefit from amplification, whereas those lacking ERPs generally experience profound hearing loss with limited speech perception abilities.

Rance et al. (2007) [163] studied 12 children with ANSD (mean age 8.5), most with mild to severe HL, all using bilateral HAs with optimal settings. These children performed below average on receptive vocabulary and articulation tests but did not differ significantly from an age- and severity-matched SNHL group.

Cochlear implantation, on the other hand, is reserved for patients who, despite adequate amplification, demonstrate unsatisfactory progress in language and verbal skill development [164].

Teagle et al. (2010) [165] proposed a stepwise management approach for children with AN, analyzing 140 cases in a prospective study. Thirty-one per cent of the children (44 out of 140) benefited from the use of HA. In the remaining cases, who subsequently received implants, the average duration of HA use prior to cochlear implantation was around 26 months. For children implanted before the age of three, the average duration was 12 months. In general, better residual hearing was associated with longer use of HAs prior to implantation, although exceptions were noted.

The American Academy of Audiology (AAA) guidelines on pediatric amplification published in 2013 [166] also established the same clinical approach; in recommendation 3 of the ‘Audiologic candidacy criteria’ section, it is advised to verify the audiometric threshold to assess speech audibility at conversational levels. Since ABR and OAE are not reliable in these cases, only careful behavioral observation allows for a precise estimate of the threshold. A trial with well-fitted hearing aids is recommended, closely monitoring the child’s responses to sounds and adjusting the amplification as necessary. Alternatively, cortical responses evoked by verbal stimuli may be used. Patients who demonstrate insufficient performance are candidates for CI.

However, the recommendation for the use of HAs in cases of HL due to ANSD has continued to be a subject of controversy. For example, a retrospective study conducted by Berlin et al. (2010) [158] reported varied outcomes regarding HA use in 85 patients diagnosed with AN. According to the results, 3.53% of patients rated their hearing aids as providing “good benefit”, 10.59% as “some benefit”, 24.71% as “little benefit”, and 61.17% as “no benefit”. In comparison, CI recipients demonstrated effective auditory rehabilitation related to speech recognition in 85% of cases, while 8% of recipients had received their implants too recently for their performance to be evaluated.

The presumed superiority of CI outcomes over HAs may also be attributed to the methodological limitations of the initial studies, which primarily included patients with severe-to-profound deafness, as highlighted by Roush et al. (2011) [167]. In these studies, 78% of the total 116 subjects had severe-to-profound HL.

A similar review conducted by Humphriss et al. (2013) [168] to evaluate the outcomes of CI in ANSD found that speech recognition ability was comparable between CI recipients (who had achieved limited results with HA) and HA users. Following CI, similar performance was observed in children with ANSD and those with SNHL. However, the authors reported that the available evidence presents several methodological limitations and therefore cannot be generalized.

An interesting experience was reported by Ching et al. (2013) [169], who conducted a longitudinal study on a cohort of 47 Australian children with ANSD and varying profiles of HL. Sixty-four percent had mild-to-severe loss, while 36% had profound loss. At the age of three, 27 children were using HA, 19 CI, and 1 was not using any assistive device. Thirty percent presented with at least one additional disability, and 80% communicated primarily through oral language. When compared with a reference population of age-matched peers with HL but without AN, no significant differences in performance emerged. However, as data from children using HAs and CI were analyzed together, it was not possible to determine any specific difficulties in children with HA. The study indicated that, in early childhood, children with ANSD can achieve levels of linguistic and auditory development that are comparable to those of their peers with other forms of HL, regardless of the device used.

A favorable effect of HAs in children with ANSD was also reported by Walker et al. (2016) [170] in a prospective multicentre study aimed at evaluating speech production, speech perception, and language outcomes. The study involved 12 children with ANSD and 22 children with SNHL, all presenting with mild-to-moderately severe SNHL and fitted with bilateral HA. The results did not reveal significant differences between the two groups regarding language and articulation outcomes. However, although children with ANSD demonstrated effective speech perception in quiet environments, a trend was observed indicating lower performance for this group, compared to those with SNHL, while using HAs in the presence of background noise.

The effectiveness of HAs (and CI) in ANSD observed in recent clinical studies over the short term appear to be sustained in the long term as well. Ehrmann-Müller et al. (2019) [73] described a cohort of children with ANSD who were monitored over time. Eight children used HA, and seventeen received CI. Acoustic amplification was implemented at an average age of 23 months. The follow-up period ranged from 4 to 14 years. Functional gain with HAs varied between 32 and 65 decibels (dB). The level of open-set monosyllabic discrimination in quiet with HAs ranged from 35% to 100%. Although this study does not report speech perception in noise, a high number of children were identified as full-time device users, a finding that reflects the subjective benefit experienced by them.

The present review indicates that HAs represent a potentially effective rehabilitative tool for many patients with AN. Although the results of diagnostic tests used to characterize the neuropathy are generally consistent across different individuals—constituting the principal marker—the severity of the disorder and response to treatment are heterogeneous among patients. This clinical variability hinders the development of standardized therapeutic algorithms. In conclusion, following the diagnostic confirmation of AN, most specialists should recommend a trial with HAs if the hearing deficit allows for the recovery of speech audibility through appropriate amplification.

#### 4.1.5. Low-Gain Hearing Aids in APD

Kuk et al. (2008) [171] were among the first to study how acoustic amplification affects people with APD. They investigated acoustic amplification in 14 children aged 7–11 years with APD and normal hearing. Participants acted as their own controls under various HA conditions, fitted with low-gain, behind-the-ear, wide dynamic range compression HAs in open-ear mode, providing approximately 10 dB of insertion gain. Using only the omnidirectional microphone, no significant speech perception improvement was found in noise. However, adding a noise reduction algorithm and directional microphones enhanced speech understanding in noise. Amplification also led to some improvement in attention in noise and on several specific questionnaire aspects. The authors suggest trial amplification in selected APD cases.

Roup et al. (2018) [172] assessed the efficacy of low-gain HAs in adults with self-reported hearing difficulties but normal audiograms. Thirty-nine participants were divided into a control group and a symptomatic group (potentially APD). Those with reported difficulties showed poorer speech recognition in noise and greater perceived hearing challenges than the controls. After a four-week hearing aid trial, the symptomatic group reported notable improvements in perceived hearing and speech-in-noise performance. Most participants tolerated the HA well, using them for one to four hours daily, although some did not experience a significant benefit. The authors conclude that low-amplification HAs may improve perceived hearing and speech comprehension in noise for selected individuals.

For a review on the potential applications of low-gain HAs in individuals with normal hearing and self-perceived auditory difficulties, see Beck et al. (2019) [109].

#### 4.1.6. Over the Counter (OTC) Hearing Aids and Others Direct-to-Consumer (DTC) Devices

In recent years, there has been a growing trend towards direct-to-consumer (DTC) approaches in the provision of healthcare services, with the aim of improving access to care and reducing costs for patients. Over the counter (OTC) hearing aids are amplification devices sold directly to consumers without a medical prescription, intended for adults with mild-to-moderate HL. Unlike personal sound amplification products (PSAPs), which amplify sounds for individuals without hearing impairment and have no therapeutic purpose, OTC HAs are regulated by the FDA [173] and are designed to enhance communication in individuals with HL. They offer self-fitting capabilities, often through dedicated applications, and are generally more affordable and accessible than traditional prescription HAs fitted by professionals. For a comprehensive review on this topic, see Manchaiah et al. (2017) [174].

There is now a growing body of evidence regarding the sustainability and efficacy of OTC HAs. In a randomized clinical trial [175] self-fitting OTC HAs with remote support were compared to audiologist-fitted devices in individuals with mild-to-moderate HL. After six weeks of use, participants in both groups reported equivalent benefits in terms of self-assessment and speech understanding in noise. Patients who were not lost to follow-up (approximately two thirds in both subgroups) were re-evaluated eight months after treatment [176] to assess the persistence and extent of the benefit. Once again, no differences in outcomes were observed between the two subgroups.

The validity of self-fitting has been demonstrated by Sabin et al. (2020) [177]. They compared a group of HA users fitted and adjusted by professionals according to best clinical practices with a group of subjects who self-fitted a commercially available OTC HA. Among the parameters customized by the patients themselves were gain and spectral profile. Scores on the APHAB and the Speech, Spatial, and Qualities of Hearing Scale (SSQ-short version) were comparable between the two groups.

Technological proficiency may influence the success of self-fitted devices. However, the proportion of individuals that are able to perform these tasks appears high (68% in Convery et al. 2019) [178], while others seem to require some form of assistance, particularly in managing the associated apps.

A special category of OTC HA is represented by pre-programmed devices. A clinical trial [154] has documented the effectiveness of this approach. Patients in one group selected a device from various pre-programmed OTC hearing aids. A comparison was made between the outcomes obtained with these devices and those fitted by a professional. Scores on two self-assessment scales, namely the Profile of Hearing Aid Benefit and the Hearing Handicap Inventory for the Elderly, were found to be equivalent between the pre-programmed OTC group and the audiologist-fitted group. Similar findings were observed in a subsequent clinical trial by some of the same authors (Humes et al. 2019) [179], using less restrictive enrolment criteria.

A meta-analysis published by Chen et al. (2022) [180] compared the effectiveness of OTC HAs (referred to as PSAPs in the text) and conventional HAs. The results indicate that OTC devices can be effective in terms of gain, listening quality, and listening effort. The authors highlight that, due to their wide availability and low cost, such devices could be considered for patients with hearing loss. Since heterogeneity can be found among different devices, it is necessary to carefully assess the characteristics of PSAPs/OTCs and conventional hearing aids when selecting options for hearing device use.

While OTC hearing devices and personal sound amplification products (PSAPs) offer a viable and cost-effective alternative to conventional HAs, their application in RAD—particularly ANSD—is problematic. Key concerns include their authorization only for adult use, despite most ANSD cases occurring in pediatric populations, and the risk that self-managed device adoption may delay the diagnosis of serious underlying conditions. Therefore, comprehensive audiological evaluation and medical clearance are essential prior to considering OTC/PSAP use. If deemed suitable by a clinician, these devices may be used in cases of adults with mild, stable HL, with appropriate support, monitoring, and regular follow-up. Ultimately, OTC/PSAP devices should only be used under specialist supervision as part of a tailored management plan. While they offer a potentially affordable and convenient option, particularly for managing the peripheral component of certain APD cases, their utility for the complex processing deficits seen in ANSD remains uncertain. Due to the lack of specialized fitting and complex signal processing necessary to address neural dys-synchrony, clinicians must exercise caution when recommending OTC devices for ANSD, emphasizing that a comprehensive diagnosis (including electrophysiological testing) is critical to rule out more complex pathologies that require prescription-based devices or cochlear implantation.

#### 4.1.7. Assistive Listening Devices (ALDs)

ALDs encompass a broad range of technologies aimed at enhancing communication for individuals with hearing loss. Unlike hearing aids, which amplify all sounds, ALDs typically deliver sound directly or process it in a targeted manner to facilitate listening in specific situations. Common examples include:

- Personal wireless systems: A remote microphone (RM) worn by the speaker transmits sound via radiofrequency (FM) or infrared (IR) to a receiver worn by the listener, thereby bypassing background noise. This approach significantly improves speech understanding in challenging environments such as classrooms or meetings.
- Telephone amplifiers and TTY/TDD devices: These support telephone conversations by amplifying the voice or converting speech to text.
- Captioned televisions and devices: Systems that display captions for TV programs or live speech, with some devices providing real-time speech-to-text conversion.
- Bluetooth and wireless streaming: Many modern HAs and cochlear implants can stream audio directly from smartphones, televisions, or other devices, functioning as ALDs to deliver clear, amplified sound without background noise.
- Alerting devices: For safety, these devices may flash light or vibrate to signal alarms, doorbells or a baby’s cry.

ALDs have particularly promising features for patients with RAD, since they can improve the signal-to-noise ratio. Indeed, the main issue for these patients is poor speech perception, especially in noisy environments. The next paragraph will examine RM technology in the context of both ANSD and CAPD, and the more general term “ALD” will be used to refer to this technology, whether used in combination with hearing aids (RMHA) or alone (RM).

#### 4.1.8. ALD in Children with APD

The usefulness of RM (personal FM system) in school-aged children with APD was demonstrated by Johnston et al. (2009) [181]. Ten children with normal hearing and evidence of APD were recruited. Prior to the use of the RM system, they showed lower speech perception scores, poorer academic performance, and more psychosocial problems compared to an age- and gender-matched control group. During the school year, prolonged use of RM led to significant improvements in speech perception in noisy environments, academic achievement, and psychosocial well-being. Furthermore, after extended use, even in the absence of the RM, progress in speech perception was observed, suggesting a possible improvement in basic auditory functions. According to the authors, intervention with RM technology is therefore effective, does not interfere with classroom teaching, may reduce the need for school support services, and represents a cost-effective solution to promote academic and social success in children with APD.

Favorable outcomes with the use of RMs in school-aged children with APD have also been reported in a randomized controlled trial [150]. Fifty-five children aged between 7 and 13 years were recruited. The indicators evaluated included parameters related to reading, language, and auditory processing. Personal RM systems provided additional benefits compared to the primary intervention, which consisted of both top-down and bottom-up auditory training.

The effectiveness of ALDs in children with APD was further assessed in a more recent trial conducted by Smart et al. (2018) [182]. Twenty-eight children aged between 7 and 12 years participated in a 20-week trial using bilateral RMs in the classroom. They were assessed with behavioral tests, cortical auditory-evoked potentials (CAEP), and questionnaires for students, teachers, and parents. The study found that personal RMs significantly improve speech perception in noisy environments for children with APDs, with positive effects being recognized by students, teachers, and parents alike. The benefits observed are mainly behavioral, while no changes were found in neurophysiological parameters.

Stavrinos et al. (2020) [183] conducted a randomized controlled clinical trial to evaluate the long-term efficacy of ALDs—specifically, hearing aids with remote microphones (RMHAs)—in children with APD. The study involved 26 children aged between 7 and 12 years, divided into an intervention group (13 children) and a control group (13 children). The intervention group used RMHAs during school lessons for 6 months, while the control group received no intervention. The intervention group reported a significant improvement in classroom listening after 3 and 6 months, according to the Listening Inventory for Education-Revised (LIFE-R) questionnaire. However, no significant changes were observed in listening abilities in noisy environments or in attention skills measured under unaided conditions. Furthermore, prolonged use of RMHAs had no negative effects on spatial listening or attention abilities. The study demonstrates that RMHAs enhance classroom listening in children with auditory processing disorder (APD) without adversely impacting spatial listening or attention skills. However, RMHAs do not yield persistent improvements in attention, indicating the necessity for their use alongside additional management approaches, such as auditory training. Clinically, RMHAs can be recommended to support classroom listening, but supplementary interventions are essential to address attention and memory difficulties. The findings underscore the importance of further research to directly compare RMHAs with other interventions and to evaluate their long-term outcomes.

#### 4.1.9. ALD in Adults with APD

It is estimated that approximately half of patients with multiple sclerosis, despite having hearing thresholds within normal limits, report listening difficulties. A study conducted by Lewis et al. (2006) [117] demonstrated, albeit in a laboratory setting, that the use of ALDs can significantly contribute to the improvement of this symptom. In the study, an RM system was used to assess speech perception in noise among patients with multiple sclerosis, comparing the results with those from a control group. Sentences were presented at 65 dB dBA via a front-facing loudspeaker, while multitalker background noise was delivered through four loudspeakers positioned at the sides of the subject. The remote microphone was placed 7.5 cm from the sentence source. Significant differences were found in the various signal-to-noise ratios between unaided listening and listening with ALDs, highlighting the effectiveness of these devices in supporting verbal comprehension under unfavorable acoustic conditions.

Koohi et al. (2017) [114] reported the results of a rehabilitation experience in a population of non-aphasic stroke survivors. Nine out of fifty patients were diagnosed with APD based on severe deficits in understanding speech in background noise, despite having normal pure-tone thresholds. These patients underwent speech-in-noise perception testing both with and without the use of an RM system. A significant improvement in the signal-to-noise ratio (SNR) of approximately 10 dB was observed with the use of RM, especially when the noise was spatially separated from the speech signal. This benefit is attributed to the enhanced neural synchronization and representation of the speech signal. RM systems have shown promise in the treatment of a significant proportion of post-stroke patients with APD who are not eligible for conventional HAs.

A prospective real-world validation of ALD treatment in adult patients with APD was conducted by Gaastra et al. (2024) [115]. They studied a group of survivors from aneurysmal subarachnoid hemorrhage (aSAH), a severe form of stroke that can lead to cognitive, psychological, and auditory deficits, including APD. The effectiveness of an ALD with wireless remote microphone technology was evaluated in 14 patients with APD, following subarachnoid hemorrhage. The Bamford–Kowal–Bench (BKB) sentences were used to assess speech discrimination in noise conditions at 60 and 65 dB, both with and without the use of the ALD. The results demonstrated a significant improvement in performance with the device: participants went from recognizing very few words without the ALD to almost all words when using it. This benefit was maintained even more than three years after the aSAH event, suggesting a lasting positive effect. As no correlation was found between cognitive deficits and the response to the device, the authors suggest that the ALD may be effective regardless of patients’ cognitive status. Furthermore, feedback collected from participants was extremely positive: the device was described as “potentially life-changing” for daily and professional life, contributing not only to improved speech comprehension but also to reduced social anxiety and fatigue.

#### 4.1.10. Cochlear Implant in AN

Cochlear hearing loss is characterized by an increase in hearing thresholds associated with a reduction in speech perception, which is strictly dependent on the degree of hearing threshold elevation. These features are related to the impairment of contractile activity of the OHC, which amplifies the vibration of the basilar membrane, thereby increasing and modulating cochlear sensitivity. The loss of the cochlear amplifier leads to the attenuation of cochlear responses, with a consequent impairment in frequency discrimination. Cochlear lesions are profoundly different from AN. The classical form of this disorder was clearly illustrated by Arnold Starr: “In 1988, I saw an 8-year-old girl with an unusual hearing disorder. The audiograms showed a mild to moderate elevation of thresholds, incongruent with the impaired speech comprehension. Her complaint was: “I can hear but not understand”” [184]. ANSD is an auditory temporal processing disorder, characterized by disruption of the temporal coding of acoustic signals in auditory fibers with consequent impairment of auditory perception dependent on temporal cues: typically speech comprehension and sound localization [4,7]. Indeed, unlike cochlear hearing loss, in which reduced audibility and frequency discrimination underlie the impairment of speech perception, in ANSD, abnormal coding of the temporal features of auditory stimuli affects speech recognition. The abnormal synchrony of auditory nerve activity also underlies the abnormalities of auditory brainstem responses (ABR), which are absent or show profound alterations despite hearing thresholds being preserved very often. In contrast, the OHC function is normal, as reflected by OAE and CMs recordings [7,8].

AN may result from lesions involving the afferent compartment of the auditory periphery at different sites, including auditory nerve fibers, inner hair cells, or their synaptic connections. Etiology encompasses genetic, infectious, toxic-metabolic, and immunological causes; however, the most well-known forms of ANSD are due to gene mutations, and the mechanisms involved are functional alterations at pre- and post-synaptic sites, including neurotransmitter release from ribbon synapses, spike initiation in auditory nerve terminals and abnormal temporal coding related to demyelination and axonal loss [7,8,185]. ANSD may present as an isolated hearing disorder or be associated with multisystem involvement. In general, isolated forms of ANSD are underlain by pre- or post-synaptic sites of lesion, whereas ANSDs with multisystem involvement are typically associated with post-synaptic damage [185].

When considering the rehabilitative options for patients with AN, the pathophysiological mechanisms underlying the disorder prevent the use of hearing aids, since sound amplification has no effect on the restoration of auditory nerve synchrony. Therefore, cochlear implants constitute the only rehabilitative tool that is potentially able to restore speech perception in patients through direct electrical stimulation of auditory fibers.

The most representative form of pre-synaptic ANSD results from mutations in the OTOF gene encoding otoferlin, a protein belonging to the ferlin family, which is involved in synaptic release [9] as well as vesicle replenishment [10] at the ribbon synapses. In this disorder, the auditory fibers are only indirectly affected, as the impairment of auditory fiber activation and conduction is induced by alterations in the amount and timing of the glutamate release. The most common phenotype consists of congenital profound deafness with an autosomal recessive pattern of inheritance [11]. Indeed, most mutations are inactivating, leading to no or little otoferlin availability, with consequent impairment of glutamate release. In these cases, the use of CI invariably restores auditory nerve activation, resulting in auditory perception recovery and language development [186]. In this regard, the outcome of CI in children affected by otoferlin-related hearing impairment is comparable to that observed in their peers with cochlear HL related to other etiologies. Recently, the advent of gene therapy is likely to replace the use of cochlear implants in the future as the rehabilitation procedure of choice [187]. Missense mutations in the OTOF gene may also result in milder phenotypes such as temperature-sensitive ANSD [7] or congenital mild-to-moderate hearing loss associated with severe impairment of speech perception and delay in language development [188]. Specifically in these latter cases, abnormal synchrony of auditory fiber discharge is deemed to result from abnormal timing of glutamate release, due to impairment of multivesicular replenishment. These patients also benefit from the use of CI, leading to the restoration of speech perception and language development [188].

The rehabilitation of post-synaptic ANSD critically depends on the site of the lesion and the associated pathophysiological mechanisms, such as degeneration of auditory fibers resulting from demyelination and axonal loss. Indeed, the effectiveness of electrical stimulation in restoring a synchronous auditory input to the CNS crucially depends on the number and functionality of the residual fibers, as well as on the distribution of lesions along the spared elements. In general, patients affected by ANSD forms involving the distal portion of auditory fibers, such as the OPA1 disease, benefit from cochlear implantation in restoring speech perception [23], whereas patients with ANSD involving the proximal portion of the auditory nerve or the entire auditory fibers show a poor outcome after cochlear implantation with little or no improvement in perceptive abilities.

**Figure 1:**
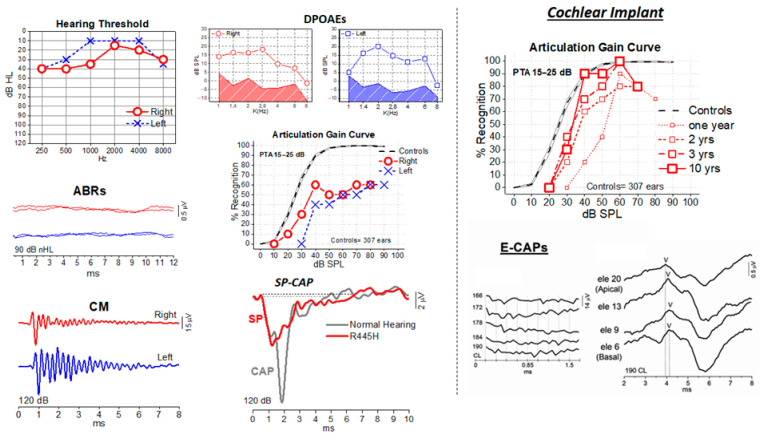
Modified from Santarelli et al. *Brain* **2015**, *138*, 563–576, with permission from [23].

A good example of successful cochlear implantation in a girl affected by OPA1 disease is shown in Figure 1. The nuclear gene OPA1 encodes the OPA1 protein, a dynamin-related GTPase, which localizes to the inner mitochondrial membrane [19,20] and is involved in several mitochondrial activities, such as fusion of the inner mitochondrial membrane, maintenance of the structure of mitochondrial cristae, and oxidative phosphorylation [188,189,190,191,192,193]). Some missense mutations affecting the GTPase domain are responsible for the syndromic form of DOA, including different extraocular manifestations, such as SNHL, ataxia, sensorimotor neuropathy, progressive external ophthalmoplegia, and mitochondrial myopathy [192].

The audiological findings from the OPA1 case, as reported in Figure 1, illustrate the profile of a post-synaptic neuronopathy [23]. The patient presented with preserved sound detection but marked difficulties in speech comprehension, low-frequency mild hearing loss, and severely reduced maximum speech intelligibility [23]. Electrophysiologically, the characteristic hallmark of AN was confirmed: preserved cochlear receptor activity (normal DPOAE, CMs, and SP) contrasted with the absence of the synchronous neural response ABR and CAP was replaced by a prolonged negative activity [23]. CI led to a remarkable and sustained improvement in speech intelligibility and perception over a 10 year period [23]. This successful outcome is hypothesized to stem from the CI’s ability to restore synchronous activation of the auditory pathways by bypassing the lesion site—the degenerated distal portion of the auditory nerve fibers. This hypothesis is supported by post-mortem human data and histological animal models, which suggest that the degeneration begins in the unmyelinated nerve terminals [193,194]. However, factors such as the progression of the underlying neurological disease and potential CNS damage must be considered, as they may lead to the deterioration of CI outcomes over time [23].

Nevertheless, although OPA1 patients can be considered good CI users, there are some factors that impact the CI outcome that should be considered. First, some patients present with deterioration of speech perception and communicative abilities over time, paralleling the progression of the neurological disease. The worsening of the disorder is likely to result in progression of the degenerative process along the auditory fibers, as well as in a decrease in spared fibers. Moreover, the occurrence of CNS damage may affect the processing of the auditory input at different levels in the auditory pathway.

When addressing the potential benefits of cochlear implantation in other forms of post-synaptic AN (see Chaudhry et al., 2020 [195] for a review), the available information appears scant and highly heterogeneous, preventing even a rough prediction of the implant outcome. First, many post-synaptic forms are associated with rare diseases or disorders with low prevalence in the general population. As a result, only individual subjects or small groups of patients affected by a specific genetic disorder are diagnosed and followed up in a medical center. In addition, diagnostic assessment is highly variable across different centers and often does not allow for the characterization of the underlying lesion. For example, the hearing disorder found in MELAS results from a mixture of cochlear and neural dysfunction, which may be present in varying proportions even among members of the same family [196]. On the other hand, as reported above, lesions localized to the proximal portion of auditory fibers are expected to have a poor outcome after cochlear implantation, due to the reduced effectiveness of electrical stimulation. In this view, the inclusion of electrophysiological recordings such as electrocochleography may help elucidate the pathophysiological mechanisms behind the hearing disorder. In conclusion, the combination of the genetic findings with the electrophysiological and audiological data may help define the site and the extent of the lesion in post-synaptic AN, thus providing crucial information for planning the rehabilitative strategy and supporting both clinicians and patients in the decision-making process.

### 4.2. Top-Down Rehabilitative Approaches

Compensatory strategies (CS) are top-down treatments used to assist individuals to overcome residual dysfunctions that interact and exacerbate auditory deficits in other areas such as language, cognition, and academic achievements. These strategies are designed to maximize the use of auditory information and include non-formal active listening to improve listening skills, auditory vigilance, verbal working memory enhancement, cognitive and metacognitive, and linguistic and metalinguistic strategies [6,90]. Improving the active listening skill is fostered through the development of awareness that listening is an active process involving self-regulation and monitoring. Active listening is a fundamental aspect of communication, characterized by an exchange in which the receiver acknowledges receipt of the information and provides feedback to the sender to ensure mutual understanding. One possible active communication approach is the “whole body listening”, a foundational concept to help make the abstract more concrete for subjects who struggle with attention, focus, and social/emotional skills [197]. Verbal working memory enhancement is generally obtained through recall of verbal information, verbal rehearsal, and filling in the gaps of a story previously read and subsequently read again with the omission of certain words [198]. Metalinguistic strategies include schema induction and discourse cohesion devices (e.g., sentences are linked and related to each other by means of references, replacement of words, lexical items, etc.), context-derived vocabulary building, phonological awareness, and semantic network expansion. Metacognitive strategies include self-instruction, cognitive problem solving, and assertiveness training.

As a matter of fact, only a single study has specifically assessed the clinical efficacy of these approaches by using evidence-based methods [199]. This randomized controlled trial aimed to determine the effectiveness of a combined protocol incorporating both auditory training—including dichotic listening exercises such as binaural integration, speech in noise tasks, and sound localization—and a compensatory strategy protocol, which consisted of whole-body listening techniques, verbal working memory enhancement, and shared reading. The outcomes were compared with those from participants who received only one of the interventions, as well as a control group who did not receive any treatment. The participants in the experimental groups performed better than the controls. In particular, the combined therapy approach had the highest average score in the speech perception and sound localization outcomes, while there were not significant differences between single AT and CS approaches, underlining the greater efficacy of combined bottom-up and top-down protocols. It is interesting to observe how CS was effective in speech perception in noise outcomes but showed no improvement in localization skills, meaning that improved attention skills in language might enable us to focus on the speaker supporting language decoding.

## 5. Conclusions

ANSD and APD are principal subtypes of RADs, distinguished by their pathophysiology and clinical implications. ANSD is defined by impaired synchronization of auditory nerve activity with preserved OHC function, whereas APD is characterized by deficits in central auditory processing despite normal peripheral hearing. Accurate differentiation is vital due to distinct etiologies, clinical presentations, and management strategies. Both conditions adversely affect speech perception, language development, and academic performance, necessitating personalized diagnostic and rehabilitative approaches.

Rehabilitation for ANSD often involves a trial with HA, which may be effective depending on the severity and nature of the neuropathy; however, clinical heterogeneity precludes standardized treatment protocols. CI outcomes are variable and depend on the site of the neural lesion, with distal involvement yielding better results. In APD, low-amplification HAs can improve speech comprehension in noise for selected cases. Over the counter and assistive listening devices may be adjunctive in APD but are of limited use in AN. AT and CS, particularly when combined, are central to APD rehabilitation and can enhance functional communication. A multidisciplinary, patient-centered approach is essential for optimal outcomes in both conditions.

As a limitation of the present study and, more generally, of narrative reviews, we acknowledge issues such as methodological reproducibility and the potential for bias arising from author judgments.

## Figures and Tables

**Table 1:** Overview of the mechanisms, clinical presentations, and genetic factors associated with distal and proximal auditory neuropathies.

	Mechanism	Clinical Presentation	Gene Involved	Ref.
Distal auditory neuropathy (dendritic/somatic post-synaptic synaptopathy)	Genetic	Optic atrophy, deafness	OPA1 codes for a mitochondrial protein that plays an important role in mitochondrial stability and energy output regulation	[19,20,22,23]
		Non-syndromic SNHL	ROR1 codes for the receptor tyrosine kinase-like orphan receptor 1, which plays an important role in the NF-κB pathway for neural outgrowth. Animal models have been correlated with deficiency of SGN axons and a lack of innervation of the sensory hair cell synapses	[24]
		CAPOS Syndrome (Cerebellar Ataxia, Areflexia, Pes Cavus, Optic Atrophy, and SNHL)	ATP1A3 codes for the α3-subunit of the transmembrane Na/K-ATPase pump, implicated in the regulation of intra- and extra-cellular ion levels	[25,26,27,28]
		Clinical and electrophysiological findings, along with the good results obtained after cochlear implantation, suggest a nonsyndromic autosomal dominant auditory neuropathy 1 (AUNA1) via a synaptic lesion, listing DIAPH3 mutations as a postsynaptic neuropathy	DIAPH3 codes for the diaphanous homolog 3, involved in cytoskeleton dynamics whose function at the synaptic and neural sites remains unclear	[29,30,31,32]
	Non genetic	Intralabyrinthine schwannoma		[33,34]
Proximal auditory neuropathy (axonal/somatic)	Genetic	Charcot–Marie–Tooth disease (CMT)	MPZ, PMP22 are both correlated to ANSD phenotype SGNs fiber demyelination	[35,36,37]
		Friedreich ataxia	SGNs fiber demyelination	[38]
		Deafness–dystonia–optic neuropathy (DDON or Mohr–Tranebjaerg syndrome) X-linked	TIMM8A deafness–dystonia peptide-1/translocase of mitochondrial inner membrane 8A (DDP1/TIMM8A) is a protein involved in the transfer of metabolites into the mitochondrial inner membrane from the cytoplasm	[39,40,41]
		Cowchock syndrome	AIFM1 codes for a flavin adenine of the mitochondrial intermembrane space, the apoptosis-inducing factor mitochondria-associated-1, expressed in inner and outer hair cells and in SGNs (delayed onset nerve hypoplasia)	[42,43,44]
		Auditory neuropathy DFNB94 and Leigh syndrome	NARS2 (SGN)	[45]
		Auditory neuropathy DFNB59 (noise induced)	Pejvakin (SGN)	[46,47,48,49,50,51,52,53]
		DFNB8 DFNB10 (AN)	TMPRSS3 The transmembrane serine protease 3 is broadly expressed in peripheral hearing pathways, notably in type II SGNs, and is involved in hair cells’ and spiral ganglion cells’ survival	[54,55,56,57,58,59,60,61,62]
		Neurofibromatosis type 2	NF2 (more of 200 alterations found) Code for Merlin, which has tumor suppressing properties	[63,64,65]
	Non genetic	Cochlear nerve deficiency (hypoplasia or aplasia)		[66,67,68]
		Dysmaturity, fetal infection (measles, mumps, CMV)		[69]
		Perinatal disorder (hypoxia with mechanical ventilation, hyperbilirubinemia, septicemia), Ototoxic drugs, Meningitis		[7,70,71,72,73,74,75]
		Thiamine deficiency		[76]
		IAC or CPA neoplasm (sporadic vestibular schwannoma, meningioma, endolymphatic sac tumors, etc.)		[77,78,79]

**Table 2:** The table illustrates specific characteristics of auditory processing, indicating the contribution of each anatomical structure within the auditory pathways by means of the symbol (+). Adapted with permission from the authors [83].

	Hair Cells	Auditory Nerve	Cochlear Nucleus	Superior Olive	Inferior Colliculus	Thalamus	Cortex
Phase locking	+++++	+++++	++++	+++	++	++	+
Neural adaptation		+	+	++	+++	++++	+++++
Gap detection		+	+	++	+++	++++	+++++
Spectral integration		+	++	+++	++++	+++++	+++++
Noise filtering			+	++	+++	++++	+++++
Spatial processing				+++++	++++	+++	+++

**Table 3:** Risk factors, clinical presentations, and mechanisms underlying APD in children and adults.

Children				
	Risk Factors	Clinical Presentation	Mechanism	Ref.
Isolated	Delayed CNS maturation or other developmental disorders Prenatal/neonatal (anoxia, hypoxia, prematurity, drug exposure, hyperbilirubinemia, CMV)	Diffuse functional deficit; not necessarily associated with any structural lesion		[1,93,99,101,102]
Associated (with neurological pathology)	Prematurity Low birth weight Epilepsy Cerebrovascular diseases Tumors Brain trauma Autism spectrum disorders	Varies based on primary diagnosis		[103]
	Genetic	There is evidence of auditory processing disorder (APD) in twin pairs. FOX syndrome auditory phenotypes	FOXG1 gene Heterozygous USH2A mutations are associated with changes in cochlear development, which may subsequently affect the development of brain regions involved in auditory processing CNTNAP2	[103,104,105,106]
Adults				
	Risk factors	Clinical presentation	Mechanism	Ref.
Isolated	Aging and exposure to noise Unmanaged APD in childhood	Peripheral and central multi-site damage	Expression of plasticity downstream of the receptor (post-synaptic) or primitive damage	[15,107,108,109]
Associated (with neurological pathology)	Tumors	Vestibular schwannoma Malignant (primary or secondary), including ependymomas and gliomas	Can induce degenerative changes in subcortical auditory pathways, especially the medial geniculate bodies and inferior colliculus, with compensatory reorganization in the auditory cortex. Neuroplasticity: The brain may attempt to reorganize auditory processing, especially in the cortex, to compensate for lost input, but this is often incomplete	[95,110,111]
	Stroke	Ischemic stroke	Bilateral common Henle’s gyrus, severe midbrain deafness, subcortical lesions are rarer, possible recovery	[112,113,114]
		Hemorrhagic stroke		[115]
	Multiple sclerosis		Fluctuating auditory disorders, tinnitus, or difficulty in understanding speech in noisy environments. Neurodegenerative diseases: Multiple sclerosis can present with sudden sensorineural HL	[116,117]
	Neurodegenerative diseases	Alzheimer’s disease		[118]
	Epilepsy			
	Infections and inflammations	Encephalitis, myelitis	Often associated with fever and systemic disorders	[119]
	Head trauma		Closed head and traumatic brain injury	[120]
	Blast injury			[121]
	Neurotoxicity	Heavy metals	Lead exposure	[122]

## Data Availability

The original contributions presented in this study are included in the article. Further inquiries can be directed to the corresponding author.

## Abbreviations

The following abbreviations are used in this manuscript:

RADs	Retrocochlear Auditory Dysfunctions
RAD	Retrocochlear Auditory Dysfunction
AN	Auditory Neuropathy
APD	Auditory Processing Disorders
ANSD	Auditory Neuropathy Spectrum Disorder
HL	Hearing Loss
HHL	Hidden Hearing Loss
ABR	Auditory Brainstem Responses
OAE	Otoacoustic Emissions
CM	Cochlear Microphonics
CNS	Central Nervous System
PTA	Pure Tone Average
MRI	Magnetic Resonance Imaging
CAEP	Cortical Auditory Evoked Potentials
IAC	Internal Auditory Canal
CPA	Cerebellopontine Angle
CI	Cochlear Implant
HA	Hearing Aid
AT	Auditory Training
CBAT	Computer-Based Auditory Training
ALD	Assistive Listening Devices
RM	Remote Microphone
RMHA	Remote Microphone Hearing Aids
OTC	Over The Counter
PSAPs	Personal Sound Amplification Products
FM	Frequency Modulation
IR	Infrared
CS	Compensatory Strategies
CMT	Charcot–Marie–Tooth disease
DDON	Deafness–dystonia–optic neuropathy
DDP1	Deafness–dystonia peptide-1
NF2	Neurofibromatosis type 2
OPA1	Gene name
ROR1	Gene name
ATP1A3	Gene name
DIAPH3	Gene name
MPZ	Gene name
PMP22	Gene name
TIMM8A	Gene name
AIFM1	Gene name
NARS2	Gene name
TMPRSS3	Gene name
CMV	Cytomegalovirus
ADHD	Attention Deficit/Hyperactivity Disorder
ASD	Autism Spectrum Disorders
ASHA	American Speech-Language-Hearing Association
AAA	American Academy of Audiology
BSA	British Society of Audiology
TTY	Teletypewriter
TDD	Telecommunication Device for the Deaf
